# Coastal Mesozooplankton Assemblages during Spring Bloom in the Eastern Barents Sea

**DOI:** 10.3390/biology11020204

**Published:** 2022-01-27

**Authors:** Vladimir G. Dvoretsky, Alexander G. Dvoretsky

**Affiliations:** Murmansk Marine Biological Institute of the Russian Academy of Sciences (MMBI RAS), 183010 Murmansk, Russia; v-dvoretsky@yandex.ru

**Keywords:** plankton, coastal waters, Novaya Zemlya, environmental influence, Arctic, Barents Sea

## Abstract

**Simple Summary:**

Arctic coastal waters have been strongly influenced by climatic fluctuations during the past decades. Recent studies reported clear warming processes in the Barents Sea and adjacent waters. Plankton assemblages are good indicators of environmental changes, and their composition and production affect all components of pelagic ecosystems. Most of data on the zooplankton in Arctic seas were obtained during summer seasons, and little is known about zooplankton communities in other seasons. Spring is one of the crucial periods in the Arctic marine environment, as primary production may reach the annual maximum. To investigate a spring pattern of Arctic mesozooplankton, we performed a study in the eastern Barents Sea. This research is the first report on the spring coastal zooplankton near the Novaya Zemlya Archipelago. We revealed high diversity and abundance of zooplankton taxa. Environmental variability had a significant impact on the mesozooplankton assemblages, with geographical location and phytoplankton density being the most important. Our data may be useful for future investigations dealing with Arctic plankton communities during the era of global climatic changes.

**Abstract:**

Mesozooplankton play an important role in Arctic shelf ecosystems as a trophic link and a key food source for many larval fish species. The distribution of mesozooplankton in the eastern Barents Sea was studied along a 500 nautical mile-long transect in May 2016 during the spring bloom. Mesozooplankton were sampled using a Juday net hauled from the surface to the bottom at 12 stations. We found good correspondence between the distribution of water masses and mesozooplankton assemblages. Mesozooplankton abundance (mean 73·10^3^ individuals m^−2^) in Novaya Zemlya coastal water was dominated by Copepoda ova and nauplii, *Thyssanoessa* spp. nauplii and *Oithona similis*. *Parasagitta elegans* and *Calanus finmarchicus* comprised most of the total mesozooplankton biomass (mean 0.9 g dry mass m^−2^) in that water mass. A second assemblage (mean abundance 171·10^3^ individuals m^−2^) was associated with the colder Barents Sea water, with *Oithona similis*, Copepoda nauplii, *Fritillaria borealis* and Cirripedia nauplii being the most numerous. In that water mass, *C. finmarchicus*, *Metridia longa*, Cirripedia nauplii and *Calanus glacialis* contributed most to the total biomass (mean 3 g dry mass m^−2^). The dominance of young stages of Copepoda and a high proportion of meroplankton were typical of spring mesozooplankton assemblages. The spatial distribution of mesozooplankton abundance and biomass was strongly correlated with latitude, longitude and chlorophyll *a* concentration, which together explained 10% of the total variance in mesozooplankton density. The present investigation is a baseline study for the assessment of the spring mesozooplankton assemblage in the eastern Barents Sea, and for an evaluation of the possible impact of future environmental changes on the Arctic shelf marine ecosystem.

## 1. Introduction

The high latitude ecosystems of the Arctic region are characterized by strong environmental seasonal and spatial variability. This is connected to high variability in recruitment of fish and shellfish stocks combined with strong biological interactions within relatively simple food webs [1,2,3,4,5]. The Barents Sea may be considered the transition zone between the boreal and true Arctic biogeographic regions. In some areas, this transition zone is very clear as a distinct polar front, but it is more gradual in other areas where the mixing of water masses occurs. The relatively warm Atlantic water that flows into the Arctic region submerges under the lighter Arctic surface water in many locations and continues as an intermediate flow into the Greenland Sea and the Arctic Ocean [6]. Most parts of the Barents Sea are strongly affected by seasonal ice. The ice cover fluctuations in the region are connected to large-scale atmospheric and oceanic circulation, the intensity of Atlantic water inflow and river runoff [6,7,8,9]. A decline in the amount of sea ice has been registered in the Arctic during past decades [10].

There are three main types of water masses in the Barents Sea, namely, Atlantic water (AW), Arctic water (ArW), and coastal water (CW). Local water masses are also formed in the Barents Sea [7]. AW is a warm saline water mass (>35.0‰) occurring mainly in the western and southwestern parts of the Barents Sea. The mean summer temperature of AW is always positive (>3 °C) [7]. AW enters into the Barents Sea from the Norwegian Sea and has a seasonal variation of approximately 1.2 °C, with the maximum in October and the minimum in March. ArW is characterized by low salinity (<34.5‰) and by temperatures below zero [8]. ArW is present mainly in the northern part of the sea. Barents Sea water (BSW) is located in the eastern basin and part of the northeastern Barents Sea and has high salinity (34.5–35.0‰) and varying temperatures (from −1.5 to +5 °C). BSW is formed primarily from AW as the result of a considerable transformation due to heat loss [6,7]. CW enters into the Barents Sea as the coastal current and is modified with river run-off, ice melt and local mixing. The salinity of CW fluctuates in a wide range, but it is usually below 34.5. Temperature of CW strongly varies (1–9 °C), depending on the area [7]. Novaya Zemlya Coastal Water (NZWC) is located approximately up to 75°N and is characterized by salinity of 33.0–34.7‰ and temperature of −1.8–+6 °C [7].

Water mass interactions and transport make the export of plankton an important feature of the Arctic region [10,11,12]. Climatic variability causes large interannual variability in ice and hydrographic conditions, which affects plankton production as well as fish and shellfish recruitment [13,14,15,16,17,18,19].

The spring bloom is one of the dominant features in the seasonal growth patterns of phytoplankton assemblage of the Arctic Ocean and adjacent shelf regions [20,21]. In nutrient-poor, high-latitude cold waters, it is usually the single seasonal peak of primary production, providing the energy and matter base for zooplankton and nekton production [8]. The spring phytoplankton bloom is the key determinant of the flow of carbon through the marine food web, and, therefore, this seasonal phase is the most important event in primary production in Arctic environment [22,23,24]. In the Barents Sea, the initial spring blooms in AW, CW and the marginal ice zone are usually dominated by diatoms [25]. These blooms start in March–April. Peaks of phytoplankton are registered in May–June when there is clear water stratification in most regions of the Barents Sea. During this period, the phytoplankton abundance tends to rise more rapidly in Polar than in Atlantic Waters [6,8,24]. The decline in phytoplankton abundance is in June–July because of rapid nutrient depletion. In the northern and northeastern parts of the Barents Sea, the spring bloom occurs later than in more southern regions, so that maximum phytoplankton abundance can be detected in June–August [7]. Towards the autumn most of the phytoplankton is comprised of microflagellates, and the total abundance tends to be decreased. During winter, phytoplankton abundance and production are low [6].

Zooplankton communities form one of the key components of biodiversity assessments, and plankton assemblages may be used as indicators to assess the status of pelagic habitat and environment in relation to climatic fluctuations [20,26,27,28,29].

The zooplankton of the Barents Sea can be divided into two large groups in accordance with main water masses. Atlantic zooplankton assemblages are transported from their core distribution and spawning area in the Norwegian Sea and include such key species as the copepod *Calanus finmarchicus*, the euphausiids *Thysanoessa inermis*, *T. longicaudata* and the hyperiid amphipod *Themisto abyssorum* [6,7,8]. The advection of zooplankton into the Barents Sea strongly affects the total pelagic secondary production in AW [7]. Arctic zooplankton assemblages are associated with cold waters located in the northern regions of the Barents Sea where the copepods *Calanus hyperboreus*, *C. glacialis*, *Pseudocalanus minutus*, *Oithona similis*, the hyperiid amphipod *Themisto libellula*, the ctenophore *Mertensia ovum* and the gastropods *Clione limacina* and *Limacina helicina* are the common taxa [6,7,8,30,31,32,33]. In general, zooplankton assemblages in the Barents Sea demonstrate clear seasonal and interannual fluctuations connected with advection of water masses, local seasonal environmental conditions, predation pressure from fish populations and climatic forcing [6,18,19,27]. Recent studies showed that the average mesozooplankton biomass in May was higher during the warmer period of 2000–2017 (7.0 g dry mass m^–2^) than during the colder period of 1987–1999 (4.6 g dry mass m^−2^) for the whole Barents Sea [17]. In the central and northern Barents Sea, marked multi-year variations of the total mesozooplankton biomass were found in August–October, and these were strongly negatively correlated with the total biomass of planktivorous fish [34].

Herbivorous copepods (especially *Calanus* spp.) contribute up to 70–90% of the mesozooplankton biomass in the Barents Sea [33,35,36], and their spawning periods are strongly associated with spring phytoplankton blooms (although some taxa (*C. glacialis*) can start to reproduce before the spring bloom using stored lipids as food sources) [37]. Smaller copepods (*Pseudocalanus* spp., *Microcalanus* spp. and *O. similis*) attain high abundances in both AW and ArW [6,38,39,40]. Their numbers vary in a wide range during the year. *Microcalanus* spp. and *O. similis* are found to reproduce during most of the year, including autumn and winter seasons, although their peaks are associated with phytoplankton blooms in spring [6,7,41].

The Novaya Zemlya Archipelago is a high-Arctic shelf system that separates the Barents Sea and the Kara Sea. The Novaya Zemlya Archipelago represents a barrier system of about 82,000 km^2^ and a maximum meridional extension of 925 km [42]. The shelf gradually deepens offshore, reaching a maximum depth of 200–230 m in the northwestern part. Two main water masses are detected near Novaya Zemlya. BSW occupies the shelf zone north of 75 °N while NZWC is present in the more southern region [7].

In spite of a number of summer zooplankton studies in the eastern Barents Sea [35,36,42,43,44], information on the spring pelagic assemblage is absent. Previous reports showed that summer mesozooplankton abundance and biomass in the coastal waters of the eastern Barents Sea varied from 47 to 851 ind m^−3^ and from 5 to 74 mg dry mass m^−3^, respectively. Copepods were the most numerous, reaching 73–98% and 61–97% of the total abundance and biomass. Two taxa (*C. finmarchicus* and *O. similis*) were the most abundant species. The mesozooplankton assemblages were related to spatial variation in environmental characteristics [35].

In general, reproduction of the major part of Arctic zooplankton is linked with either the maximum of phytoplankton production or 1–4 weeks later [6,7,20,31]. Young stages and nauplii of common copepods as well as meroplankton can be used as indicators of spring zooplankton assemblages [6,8]. In southern and western regions, the peaks of meroplankton and younger stages of copepods are found in late March–April, while these groups reach their maximum density in May–June in the central and eastern regions and in July–August in the northern areas [6,7]. Therefore, we can hypothesize that the mesozooplankton in the eastern Barents Sea would have spring features, including the presence of young copepod stages and meroplanktonic groups. Our study allowed for expanding our knowledge on the mesozooplankton in the Barents Sea and could be used as reference data for future comparisons with other studies covering other seasons in the Arctic marine environment.

Considering the fact that the response of the Arctic plankton community to oceanic warming must be recognized when data for all season are available, we performed a study to investigate a mesozooplankton pattern near Novaya Zemlya during the spring phytoplankton bloom. The aim of this paper is to (1) provide baseline data on the spring zooplankton in a less-studied and hard-to-reach Arctic region, and (2) examine spatial differences in mesozooplankton assemblage in the eastern Barents Sea with respect to environmental conditions.

## 2. Materials and Methods

**Figure 1:**
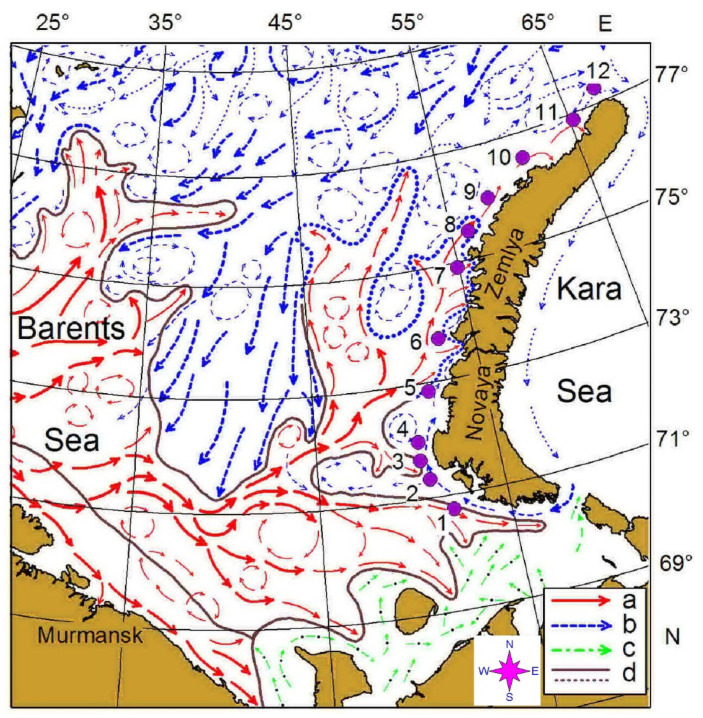
Location of the sampling area in the eastern Barents Sea in May 2016. Location of main currents and frontal zones according to [45]: a—warm currents; b—cold currents; c—coastal currents; d—boundary of the frontal zones.

Our survey was conducted west of the Novaya Zemlya Archipelago in the eastern Barents Sea, on board R.V. *Dalnie zelentsy*, in May 2016 (Figure 1, Table 1). It consisted of 12 predefined stations along a transect (Figure 1) oriented south–north between 70°45′ N and 77°17′ N. Along this transect, mesozooplankton samples were collected in triplicate at each station. The transect crossed the Polar Front zone between stations 6 and 7 where the warm water of Atlantic origin interacted with colder Arctic waters (Figure 1). Stations 1–6 were located in Novaya Zemlya Coastal Water while the rest of the stations were situated in the Barents Sea Water.

Hydrographic data were collected using a conductivity–temperature–depth (SeaBird SeaCat SBE-19, CTD) profiler. At each sampling site, water for chlorophyll *a* analysis was collected using 5 L Niskin bottles attached to a CTD rosette (10 bottles). The size-fractionated chlorophyll *a* concentration was determined for samples passed sequentially through 0.6 µm Vladiopore filters. The filters were kept frozen until analysis in a hydrochemical laboratory. The filters were subsequently extracted in 90% acetone, placed in a freezer at 4 °C for 24 h, and chlorophyll *a* concentrations were measured using a Nicolett Evolution 500 spectrophotometer (Spectronic Unicam, Cambridge, UK) which had been calibrated with commercially purified chlorophyll *a* preparations [24].

Mesozooplankton samples were taken with vertical hauls from near the bottom up to the surface. A Juday net (180 µm mesh; 38 cm diameter; 0.11 m^2^ mouth opening) was used. Assuming the entire column was filtered, no flowmeter was used to estimate volume. The volume filtered was calculated by multiplying the distance traveled by the net and the net mouth area. We assume a filtration efficiency of 100% in accordance with other studies and standard procedures suggested for zooplankton sampling in the Barents Sea [7,26,27,38,46,47,48,49]. Samples from the cod-end were preserved in 4% buffered formalin–seawater solution for taxon identification. In the laboratory, samples were split (using a pipette splitter) so that at least 400–500 organisms were in the sub-sample. Most zooplankton organisms were identified to species level and counted under an MBS-10 stereomicroscope at 4×–16× magnification using a Bogorov tray. Subsamples were no less than 1/10 of the total sample. Abundance was expressed as individuals m^−2^ or as individuals m^−3^ to compare our data with previous estimations presented using one of these units. All mesozooplankton were sorted, counted and identified to the lowest possible taxon. Developmental stages of *Calanus* species were identified by prosome length [50]. For each taxon counted, biomass estimates were made using published mean individual wet, dry or carbon weights and length/weight relationships [51,52,53,54,55,56,57,58,59,60]. All values were computed as mg dry mass (DM) per square meter using the relationship: 1 mg wet weight = 0.2 mg dry weight = 0.1 mg C [61]. The conversion of wet to dry weight for Ctenophora and Hydromedusae assumed 1 mg wet weight = 0.04 mg dry weight [61].

We used abundance data expressed as individuals per square meter instead of individuals per cubic meter in statistical analysis because the sampling depth varied by a wide range in our study. Therefore, we had the opportunity to compare our values with previous data obtained in shallow and deepwater regions.

The structure of the mesozooplankton assemblage was examined by multivariate techniques using the PRIMER software package [62]. Copepoda ova and Copepoda nauplii were included in the statistical analyses as combined groups because they had high relative abundance in the total mesozooplankton. Additionally, the presence of Copepoda ova and nauplii is an indicator of the spring state of the mesozooplankton assemblage in the region. A classification procedure was employed to group samples with similar composition. Prior to analysis, all abundance data were transformed using the square-root transformation. Cluster analysis was based on the Bray–Curtis similarity measure and group average linkage classification [62]. The percentage contribution of each species to within- and between-group dissimilarity was determined using the similarity percentages (SIMPER) procedure within the PRIMER software with an analysis of similarity (ANOSIM) procedure to test for variation in the zooplankton composition between the survey sites. Differences in environmental parameters and mesozooplankton between groups were tested using one-way ANOVA. Assumptions of ANOVA were checked using a Kolmogorov–Smirnov test for normality and a Levene test for homogeneity of variances. If the data were not normally distributed, a Kruskal–Wallis test was performed. Pielou evenness (J) and Shannon diversity indices (H’) [63,64] were calculated in order to assess species diversity in each station.

Possible relationships between the most abundant mesozooplankton taxa/groups and environmental variables were explored using canonical correspondence analysis (CCA) with the Canoco 5.0 software package [65]. Prior to analysis, species abundances were log_10_(x + 1)-transformed and those present at only one site were removed from the data matrix. A total of six environmental variables (latitude, longitude, depth, mean water temperature, mean salinity and mean chlorophyll *a* concentration) were included in preliminary analyses. The forward selection option in Canoco was then used to identify environmental variables which significantly affected the distribution (*p* < 0.100, Monte Carlo permutation test). Latitude and longitude were included in the CCA because these variables reflect the geographical positions of stations and may indirectly indicate an influence of water masses on the mesozooplankton. In addition, correlations between mesozooplankton abundance/density of common taxa and environmental factors were determined using Spearman’s rank correlation analysis. A Holm–Bonferroni statistical correction was applied to the correlations to control the probability of a type I error with multiple comparisons.

## 3. Results

**Figure 2:**
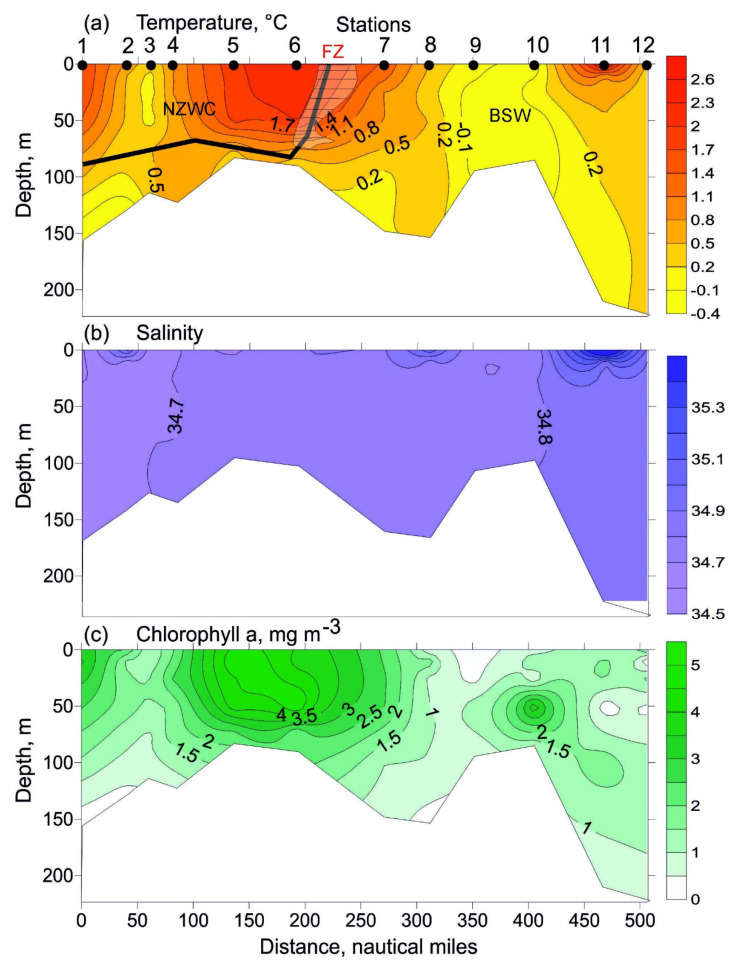
Vertical profiles of temperature ((**a**), °C), salinity (**b**), and chlorophyll *a* concentrations ((**c**), mg m^−3^) along the transect sampled in the eastern Barents Sea in May 2016. Bold line indicates boundaries between different water masses: NZWC—Novaya Zemlya Coastal Water; BSW—Barents Sea Water; FZ—location of a thermal frontal zone. Chlorophyll *a* data are obtained from [24].

Near-surface temperature ranged from −0.3 °C (station 10) to 2.3 °C (station 6). South of station 7 there was a marked horizontal gradient (T = 1.0 °C) (Figure 2a). Bottom temperature varied from −0.4 °C (station 2) to 0.7 °C (station 4). Mean values are presented in Table 1. Surface salinity increased from 34.64 to 35.00 north of station 3 (Figure 2b). Bottom salinity was stable, varying in a range of 34.67–34.89. The resulting temperature and salinity distribution (Figure 2a,b) showed little stratification across the entire study area, with a weak thermocline below 70 m at stations 1, 2, 6, 7 and 11.

The chlorophyll *a* concentration in the surface layer at the southern part of the transect (stations 1 and 2) was generally > 2.5 mg m^−3^. Surface concentrations from 1.1 to 1.9 mg m^−3^ were found at stations 3 and 4 [24]. Maximum chlorophyll *a* concentrations (4.0–4.6 mg m^−3^) were recorded in the surface layer in the central part of the transect (stations 5 and 6). High chlorophyll *a* concentration (2.4 mg m^−3^) was also found at station 7. Surface chlorophyll *a* concentration decreased north of this location, with the minimum value (0.21 mg m^−3^) at station 9 [24]. The northernmost station was characterized by higher chlorophyll *a* concentration (2.1 mg m^−3^). A similar pattern was observed for chlorophyll *a* distribution in the 10–50 m layer (Figure 2c). Bottom chlorophyll *a* concentration ranged from 0.18 mg m^−3^ (station 1) to 2.50 mg m^−3^ (station 6) [24]. A phytoplankton bloom occurred across the study area, with high proportions of centric diatoms (*Thalassiosira* spp.) and pennate diatoms. The prymnesiophyte *Phaeocystis pouchetii* was abundant north of station 6 (E.I. Druzhkova, personal communication).

A total of 54 mesozooplankton taxa/groups were found in the samples (Table 2).

**Figure 3:**
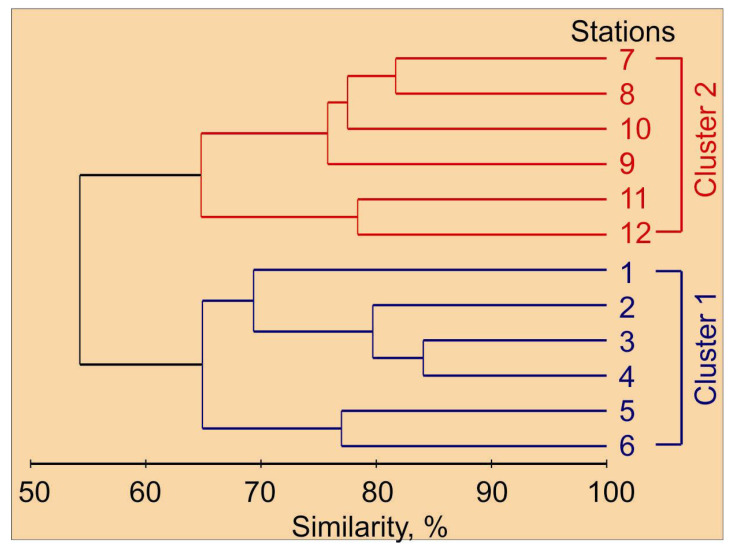
Percent similarity cluster dendrogram of mesozooplankton abundance (individuals m^−2^) in the eastern Barents Sea in May 2016.

Holoplankton organisms were the major constituent of mesozooplankton taxa, despite the high relative abundance of meroplankton organisms (cirripedia, echinodermata and gastropoda larvae). Cluster analysis on the mesozooplankton species abundance matrix allowed us to delineate two major groups of stations (Figure 3). These clusters matched the zonation based on hydrographic conditions. Cluster 1 corresponded with the Novaya Zemlya coastal water, while Cluster 2 was related to the Barents Sea water, where colder waters were found (Table 1 and Table 2).

In Cluster 1, *Thyssanoessa* spp. nauplii (24 ± 6%), Copepoda ova (21 ± 5%), Copepoda nauplii (18 ± 4%) and *Oithona similis* (14 ± 4%) were the dominant groups in terms of total mesozooplankton density (Table 2). Cluster 2 was defined by the dominance of *O. similis* (18 ± 4%) coupled with relatively high abundances of Copepoda nauplii (17 ± 5%), *Fritillaria borealis* (12 ± 3%) and Cirripedia nauplii (11 ± 3%). Mesozooplankton biomass at Clusters 1 and 2 ranged from 375 to 1485 mg dry mass m^−2^ and from 1001 to 9591 mg dry mass m^−2^, respectively. *Parasagitta elegans* (32 ± 9%) and *C. finmarchicus* (20 ± 9%) contributed mostly to the total mesozooplankton biomass at stations of Cluster 1, while Cluster 2 was dominated by *C. finmarchicus* (30 ± 3%), *M. longa* (17 ± 7%), Cirripedia nauplii (13 ± 6%) and *C. glacialis* (12 ± 1%).

**Figure 4:**
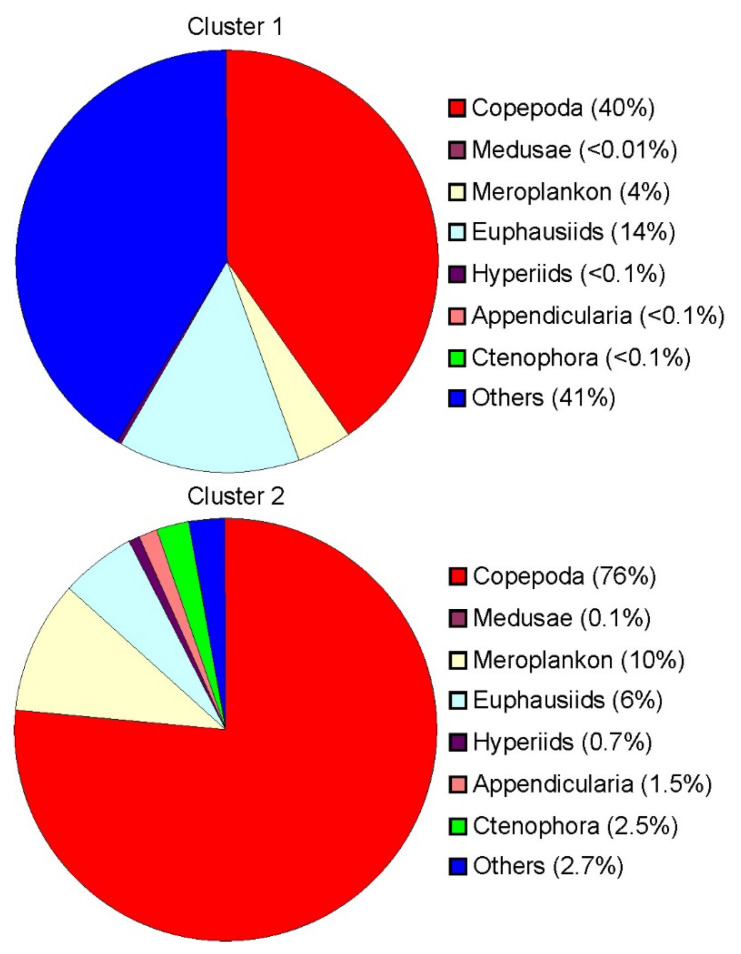
Relative biomass of mesozooplankton in each group defined by cluster analysis in the eastern Barents Sea in May 2016.

The mesozooplankton assemblage at stations of Cluster 1 was different from the one at stations of Cluster 2 at the 55% similarity level (ANOSIM, R = 0.78, *p* = 0.002). The clusters differed significantly in abundance of *C. glacialis*, *C. hyperboreus*, *M. longa*, *Microcalanus* spp., *Pseudocalanus* spp., *Chionoecetes opilio* larvae, *Thyssanoessa* spp. nauplii, *F. borealis* and *Oikopleura* juveniles (Table 2). Subsequent SIMPER analysis revealed that the taxa contributing most to the separation of the two groups of stations were *O. similis* (15%), Copepoda nauplii (13%), *F. borealis* (13%), *Microcalanus pygmaeus* (12%), Cirripedia nauplii (12%) and *Thyssanoessa* spp. nauplii (11%). Clusters differed significantly in total mesozooplankton abundance, biomass and diversity. Mean water temperature, salinity and chlorophyll *a* concentration were also significantly different between station groups (Table 2). The contribution of main mesozooplankton groups to the total biomass at each cluster is shown in Figure 4.

**Figure 5:**
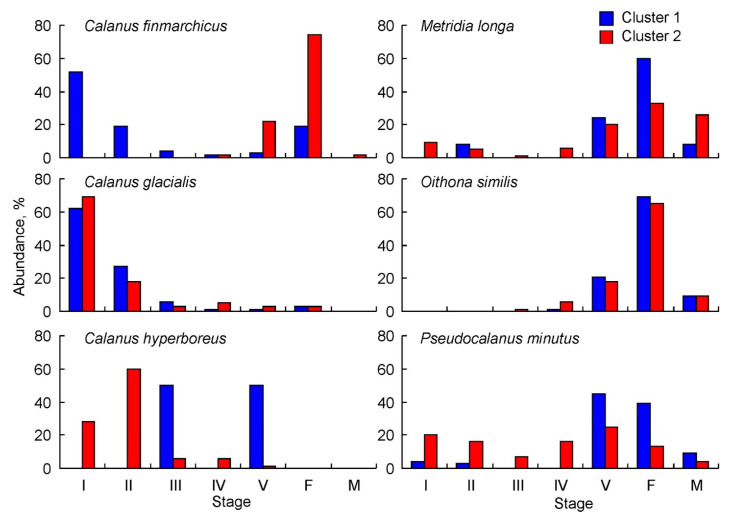
Population structure of common copepod taxa in the eastern Barents Sea in May 2016. I–V—copepodites I–V; F—female; M—male.

For *C. finmarchicus,* young copepodite stages dominated by CI–CII copepodites constituted 71 ± 10% at stations of Cluster 1, and copepodites CV (22 ± 4%) and females (74 ± 4%) were most numerous at stations of Cluster 2 (Figure 5). *C. glacialis* had the highest abundance of CI (62 ± 4% in Cluster 1 and 69 ± 3% in Cluster 2) and CII copepodites (27 ± 3% in Cluster 1 and 18 ± 3% in Cluster 2) (Figure 5). A similar pattern was found for *C. hyperboreus* at stations of Cluster 2, where CI–II copepodites contributed 88 ± 28% of the total population density. In contrast, copepodites CIII (50 ± 29%) and CV (50 ± 29%) were only recorded at stations of Cluster 2 (Figure 5). *M. longa* were predominately CV copepodites and adults (92 ± 15% in Cluster 1 and 79 ± 16% in Cluster 2). Young copepodite stages contributed little to the total *M. longa* abundance (9–14%) (Figure 5). *O. similis* was present mainly as old copepodites, but its abundance was underestimated due to the coarse net used for sampling (Figure 5). *Pseudocalanus* spp. populations were dominated by older stages (CV–adults, 93 ± 15%) at stations of Cluster 1, while younger stages (CI–CIII, 43 ± 16%) prevailed at stations of Cluster 2 (Figure 5).

**Figure 6:**
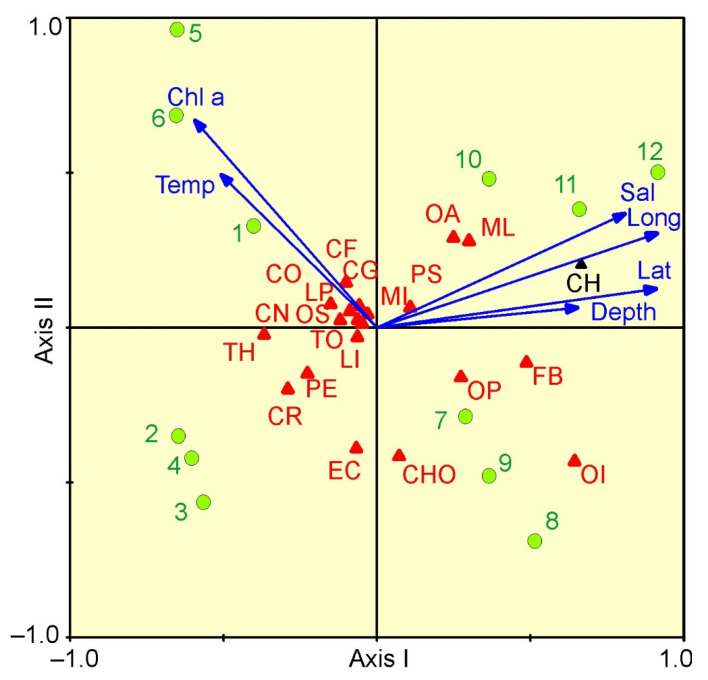
CCA for the most abundant mesozooplankton taxa/groups and environmental parameters of samples collected in the eastern Barents Sea in May 2016: CF—*Calanus finmarchicus*; CG—*Calanus glacialis*; CH—*Calanus hyperboreus*; CO—Copepoda ova; CN—Copepoda nauplii; ML—*Metridia longa*; MI—*Microcalanus* spp.; OA—*Oithona atlantica*; OS—*Oithona similis*; PS—*Pseudocalanus* spp.; CR—Cirripedia nauplii; EC—Echinoidea (echinopluteus larvae); OP—Ophiuroidea (ophiopluteus larvae); LP—Polychaeta larvae; CHO—*Chionoecetes opilio* larvae; LI—*Limacina helicina*; PE—*Parasagitta elegans*; TH—*Thyssanoessa* spp. larvae; FB—*Fritillaria borealis*; OI—*Oikopleura* juv.; TO—total. Lat—latitude; Long—longitude; Depth—depth of sampling (m); Temp—mean temperature (°C); Sal—mean salinity; Chla—surface chlorophyll *a* concentration (mg m^–3^). Green circles indicate sampling stations.

The relationships between mesozooplankton abundance and environmental variables are presented in a CCA biplot (Figure 6). The Monte Carlo permutation test indicated significance in the ordination diagram (F ratio = 2.83, *p* < 0.001) in which the first two axes explained 70.3% of the total variance. From CCA, the first axis (eigenvalue 0.061; 49.0% in total species-environment relation) was strongly positively related to latitude, longitude and salinity, and negatively related to chlorophyll *a* (Figure 6). The second axis (eigenvalue 0.026; 21.3% of total variance) was negatively related to chlorophyll *a* concentration. *C. hyperboreus*, *M. longa* and *F. borealis* were related to the first axis, and their abundances increased with latitude and longitude (Figure 6).

Most of the herbivorous/omnivorous taxa were related to the high chlorophyll *a* values characterizing the stations of Cluster 1. The forward selection of environmental factors with Monte–Carlo permutation tests (999 permutations) revealed that latitude, longitude and chlorophyll *a* concentration were the significant factors that contributed to the observed variability in mesozooplankton abundance. The three environmental variables together explained 10% of the total variance in mesozooplankton density.

Correlation analysis showed that total mesozooplankton abundance was positively correlated to longitude and negatively to mean temperature and chlorophyll *a* concentration (Table 3). The abundance of *C. glacialis*, *C. hyperboreus*, *M. longa*, *Microcalanus* spp., *Pseudocalanus* spp. and *F. borealis* increased with latitude/longitude and decreased with increasing temperature (Table 3). Abundance of common mesozooplankton taxa was negatively correlated to the surface chlorophyll *a* concentration (Table 3).

## 4. Discussion

The present study has provided novel information on the relationships between mesozooplankton distribution and hydrographic variables in the eastern Barents Sea during spring bloom conditions. Prior expeditions focused only on the summer mesozooplankton assemblages [26,36,42,43,44]. The results discussed here can provide support for future investigations on the mesozooplankton biology in Arctic pelagic ecosystems.

In May 2016, the water column off the Novaya Zemlya Archipelago was well mixed, with higher temperatures in the southern part of the study area. Novaya Zemlya coastal water was present at stations 1–7, where temperature was in accordance with typical hydrology described earlier for the eastern Barents Sea [7]. The latter stations were colder, indicating the presence of BSW, which are characterized by the lower temperatures [7]. The two water masses were separated with a thermal front located between stations 6 and 7, where the difference in the mean temperature was 1.1 °C. Therefore, temperature was the factor delineating stations by their hydrological features. Such a pattern is expected and can be found in other regions of the Barents Sea where different water masses interact [6,8,27,38,39]. Salinity was high in the study area and did not show any spatial differences. This salinity pattern suggested a strong influence of Atlantic waters, which interfaced with the general water circulation in the Barents Sea (see Figure 1). Compared to mean spring temperatures in previous years (1930s–2001), temperatures were higher in May 2016 by 1.0–1.5 °C [66]. Therefore, our study period may be characterized as a warm spring.

Chlorophyll *a* values in the Novaya Zemlya Coastal Water were higher compared to the values from the central Barents Sea (1–2 mg m^−3^; Atlantic and Polar Front waters) during spring bloom [25] and from the northern Barents Sea (0.1–3.0 mg m^−3^; Arctic waters, early bloom) [67]. In addition, Wassmann et al. (1999) reported that chlorophyll *a* concentrations in the marginal ice zone were higher than 6 mg m^−3^ at 0–30 m [25]. Values in the spring of 2016 [24] were also higher compared with chlorophyll *a* concentrations recorded near Novaya Zemlya during the summer and autumn periods, when they did not exceeded 0.2–2.0 mg m^−3^ [45]. This discrepancy might be due to changes between seasons. The spring bloom in arctic ecosystems usually starts with an early phase characterized by low phytoplankton density and chlorophyll *a* concentration [8,21]. The early phase continues into a second phase: the growth phase dominated by larger phytoplankton, such as *Phaeocystis pouchetii* colonies and a few diatom genera [68]. The maximum spring bloom, with dominating centric and chain-forming diatoms and high phytoplankton biomass/chlorophyll *a* concentration, follows the growth phase [3,6,25]. After the vernal bloom, an oligotrophic phase occurs with flagellated forms and low total phytoplankton biomass [68]. In our case, a bloom phase of phytoplankton succession was observed within the study area. Despite patchiness in chlorophyll *a* distributions, there was a difference in the mean values in NZCW and BSW. This pattern can be explained by phytoplankton succession in warm and cold waters as well as nutrient concentrations and zooplankton grazing [24]. Previous studies conducted in the central Barents Sea have shown that in the polar front and ArW, spring bloom started earlier than in AW [6,25]. This pattern may be connected with stronger water column stratification and stability in the polar front and ArW than in AW, leading to enhanced nutrient concentrations that promote earlier growth of phytoplankton species and shift the start of a classical spring bloom in ArW and the marginal ice zone [25]. Our study also suggests that phytoplankton growth begins later in the NZWC in comparison to BSW.

The composition and distribution of mesozooplankton species/taxa found in our sampling were in general similar to results previously obtained in the eastern Barents Sea in summer seasons [35,36,42,43,44], with copepods being the most diverse and numerous group. Comparisons with other studies performed in the other regions of the Barents Sea in the spring periods have shown good accordance. For example, copepods (*O. similis*, *C. finmarchicus*, *C. glacialis*, *M. longa*, *Pseudocalanus* spp., *Microcalanus* spp. and Copepoda nauplii) dominated mesozooplankton abundance in the central Barents Sea in May 1998 [32]. Similar results were reported for the central Barents Sea in May 1999 [31]. The faunal similarity in our study (Bray–Curtis indices) was >50%, which suggests stability in mesozooplankton composition around the archipelago. This pattern is associated with a local eddy existing around Novaya Zemlya [42]. Up to 72 taxa can be found in zooplankton samples from the eastern Barents Sea [69]. In our samples, we found more than 50 taxa and higher Shannon indices of mesozooplankton than in other Arctic regions in summer seasons [14,38,46,70,71]. This suggests high diversity of mesozooplankton assemblages near the Novaya Zemlya archipelago in spring season. This result is expected because spring zooplankton assemblages include more meroplankton taxa, which enrich fauna of the regions during this period.

The study showed the influence of the two main water masses off the Novaya Zemlya Archipelago on the abundance, biomass and common members of the mesozooplankton communities. The first assemblage, associated with the local water mass (NZWC), was characterized by higher proportions of boreal taxa relative to the assemblage recorded in BSW. For instance, *C. finmarchicus* accounted for 5% of the total abundance in Cluster 1, while its relative abundance was 2% at stations of Cluster 2. The second assemblage in the more northern part of the study transect was characterized by slightly higher proportions of Arctic taxa (e.g., combined relative abundance of *C. glacialis* and *C. hyperboreus* was 2 and 4% in NZWC and BSW, respectively). We also found *C. finmarchicus* reproducing in the coastal waters near the southern part of Novaya Zemlya, while there were no younger stages of this species in the north of the study area, probably because of less-favorable temperature conditions for boreal zooplankton taxa. Strong relations between zooplankton distribution and hydrographic conditions (water masses, circulation pattern and local eddies) have been reported previously for the eastern Barents Sea [35,36] and other Arctic regions [5,38,39,46,47,48,49,72,73,74].

The mesozooplankton assemblage located off the Novaya Zemlya archipelago had typical spring features, with a dominance of meroplankton, larval stages of crustaceans and younger copepodites of *Calanus* spp. In the present study, these groups accounted for more than 40% of the total mesozooplankton abundance, especially in the southern part of the study area. In high latitudes, zooplankton respond to the initial phytoplankton spring bloom by increasing abundance more than biomass due to stimulated reproduction of overwintering adult copepods [5,13,71]. A lag in the grazing response of herbivorous zooplankton at the beginning of the bloom promotes rapid phytoplankton accumulation [8]. Higher phytoplankton concentration then stimulate grazing by overwintering stages. Lower chlorophyll *a* concentrations and phytoplankton composition recorded in BSW in our investigation suggest that spring bloom began earlier in the northern part of the study transect. Temporal development of zooplankton assemblages in the Barents Sea is mainly controlled by the seasonal succession of phytoplankton [6,7,8,75]. Maximum zooplankton abundance in the Barents Sea may be observed during bloom conditions, or it can be detected after phytoplankton peak with a lag of 2–4 weeks [7,14].

Mesozooplankton abundance values recorded during our study were 4 to 8 times higher than in previously published summer studies [35,42], whereas biomasses were 1.2–2 times lower. Considering similar methods to calculate mesozooplankton abundance and biomass, we may hypothesize that strong discrepancies with previously recorded abundance may arise from differences in sampling periods. According to the seasonal pattern of zooplankton production in Arctic shelf ecosystems [6,7,42,75], our biomass estimations were intermediate between maximum late spring values and winter minimum values.

The results of a correlation analysis and CCA between biological and environmental variables indicated that geographical location, water temperature and chlorophyll *a* concentration were the main factors determining mesozooplankton distribution across the study area. As expected, the abundance of Arctic mesozooplankton taxa tended to increase with a decrease of water temperature, reflecting their preference for colder water. Similar data have been reported for summer mesozooplankton assemblages in the eastern and central Barents Sea [35,38], Svalbard coastal waters [13,71,72] and in the northern White Sea [76]. Recent studies suggest that climate fluctuations may impact whole pelagic ecosystems from micro-producers and zooplankton to higher trophic levels [17,19,27,75,77,78]. For instance, in a climatically cold year, the plankton biomass was highest in the Arctic waters of the northeastern Barents Sea because of an increase in abundance of large Arctic amphipod species. In a climatically warm year, the zooplankton biomass was high in the Atlantic waters of the southwestern Barents Sea. This pattern was due to a higher inflow of advected organisms and high temperatures, which may have resulted in the accelerated growth of zooplankton [17,19]. In our study, we revealed a negative correlation between mesozooplankton abundance/biomass and chlorophyll *a* concentration. Such a pattern may have been associated with local circulation near Novaya Zemlya, which resulted in patchiness in the plankton distribution and impacted grazing by herbivorous mesozooplankton. In particular, our data showed higher mesozooplankton density and lower chlorophyll *a* concentration in the colder waters (BSW, Cluster 2) compared to warmer waters (NZCW). This pattern suggests earlier phytoplankton development in the northern part of the study area, where grazing of a dense herbivorous mesozooplankton caused lowered phytoplankton biomass. Therefore, our data is in accordance with some previous studies in the central and western Barents Sea, where phytoplankton growth and a consequent burst of mesozooplankton organisms were earlier in colder waters of ArW and the polar front relative to warmer waters of AW [6,7,30,31].

## 5. Conclusions

Although our dataset was limited by a short period and 12 stations, we were able to obtain novel data on the Arctic marine zooplankton during the spring season. The present investigation is the first report on the spring mesozooplankton assemblage in the eastern Barents Sea and should be considered as a baseline study for the assessment and evaluation of the possible impact of future environmental changes on the Arctic shelf marine ecosystem. The climate of the Barents Sea and other Arctic regions is changing, as revealed by increasing temperatures associated with Atlantic water inflow and reduced ice cover. The impact from climatic changes may take effect as a bottom-up cascade from micro-producers to mesozooplankton and plankton-feeding fish. Predicted variations in temperature could change the timing of phytoplankton blooms and, therefore, alter the structure of zooplankton assemblages, carbon cycling in pelagic ecosystems and food availability for higher trophic levels on the Arctic shelf. Summarizing the main results of our study we can conclude the following:

Mesozooplankton demonstrated spatial variability in abundance and biomass across the range of environmental conditions within the study area. The high density of meroplankton and younger stages of copepods clearly indicated a spring state of the mesozooplankton assemblage.

Two mesozooplankton assemblages were delineated using hierarchical clustering based on the abundance data.

These assemblages demonstrated a strong association with water mass distribution. Higher values of total abundance and biomass were recorded in the northern part of the study area, where the earlier peak of phytoplankton supported a more diverse and abundant mesozooplankton assemblage.

Canonical correspondence and correlation analyses revealed geographical location, water temperature and chlorophyll *a* concentration to be the main factors affecting mesozooplankton abundance across the study area.

## Figures and Tables

**Table 1:** Properties of the stations visited in the eastern Barents Sea in May 2016.

ID	Date (May 2016)	Latitude (N)	Longitude (E)	Local Time	Depth, m	Temp	Sal	Chla	Ab	Biom
1	10	70°45′	51°60′	8:10	156	0.9	34.66	2.35	84/597	0.65/5
2	10	71°20′	51°00′	13:45	130	0.2	34.67	1.49	126/968	1.49/11
3	10	71°40′	50°39′	18:18	114	0.3	34.66	1.15	74/741	1.06/11
4	10	71°60′	50°41′	21:30	122	0.7	34.70	1.76	82/745	1.1/10
5	11	72°51′	51°43′	19:20	85	1.6	34.74	4.11	42/531	0.41/5
6	12	73°44′	52°50′	2:05	90	1.7	34.78	3.58	29/364	0.38/5
7	12	74°55′	54°54′	12:35	149	0.6	34.77	1.95	134/954	1.23/9
8	12	75°30′	56°07′	19:21	169	0.4	34.78	0.92	133/833	1.00/6
9	13	76°00′	57°55′	0:20	94	−0.2	34.74	0.53	271/3009	1.79/20
10	13	76°32′	61°01′	7:32	86	−0.2	34.77	1.57	108/1345	1.18/15
11	13	76°53′	65°17′	15:45	227	0.3	34.90	1.23	195/929	2.82/13
12	13	77°17′	67°30′	20:27	231	0.3	34.88	1.04	188/819	9.59/42

Temp—mean temperature (°C); Sal—mean salinity; Chla—mean chlorophyll *a* concentration (mg m^−3^) [24]; Ab—mesozooplankton abundance (10^3^ individuals m^−2^/individuals m^−3^); Biom—dry mesozooplankton biomass (g m^−2^/mg m^−3^). Note that abundance and biomass data are presented in terms of area (per m^−2^) and in terms of water volume (per m^−3^), respectively.

**Table 2:** Mean (individuals m^–2^ with standard error) abundance of mesozooplankton taxa; evenness (J); diversity (H’, Shannon index); total mesozooplankton abundance (10^3^ individuals m^–2^); total mesozooplankton biomass (mg dry mass m^–2^); environmental parameters (Temp—mean temperature, °C; Sal—mean salinity; Chla—mean chlorophyll *a* concentration, mg m^–3^) in each group defined by cluster analysis and results of comparisons between groups (simple ANOVA or Kruskal–Wallis Test).

Taxon/Parameter	Trophic Status	Biogeography	Cluster 1	Cluster 2	ANOVA or Kruskall–Wallis Test (*p*-Level)
**Copepoda**			49,086 ± 14,379	118,331 ± 32,689	<0.05
*Calanus finmarchicus* (Gunner, 1765)	He	Bor	2472 ± 567	3243 ± 1225	0.581
*Calanus glacialis* Jaschnov, 1955	He	Ar	1925 ± 430	5396 ± 1485	<0.05
*Calanus hyperboreus* Krøyer, 1838	He	Ar	6 ± 5	2400 ± 1518	<0.05
*Centropages hamatus* (Lilljeborg, 1853)	Om	Bor-Ar	34 ± 24	14 ± 10	0.818
Copepoda ova		Bor/Bor-Ar	13,315 ± 2602	8308 ± 1234	0.113
Copepoda nauplii	Om	Bor/Bor-Ar	13,263 ± 4304	27,933 ± 8075	0.140
*Gaetanus tenuispinu* (Sars G.O., 1900)	Om	Ar	14 ± 11	6 ± 4	0.937
*Metridia longa* (Lubbock, 1854)	Om	Ar	122 ± 52	7039 ± 5037	<0.05
*Microcalanus pusillus* Sars G.O., 1903	Om	Bor-Ar	312 ± 111	1473 ± 282	<0.05
*Microcalanus pygmaeus* (Sars G.O., 1900)	Om	Ar	5433 ± 2239	22,941 ± 2287	<0.001
*Microsetella norvegica* (Boeck, 1865)	Om	Cs	20 ± 9	9 ± 9	0.485
*Oithona atlantica* Farran, 1908	Om	Bor	31 ± 15	239 ± 90	0.093
*Oithona similis* Claus, 1866	Om	Cs	11,884 ± 3925	33,218 ± 8691	0.132
*Triconia borealis* (Sars G.O., 1918)	Om	Cs	-	67 ± 48	na
*Parathalestris croni* (Krøyer, 1842)	Om	Bor-Ar	3 ± 3	5 ± 5	0.937
*Pseudocalanus spp.* I–IV	He	Bor-Ar	33 ± 33	3933 ± 1991	<0.05
*Pseudocalanus minutus* (Krøyer, 1845) V–VI	He	Bor-Ar	152 ± 32	1767 ± 626	<0.05
*Pseudocalanus acuspes* (Giesbrecht, 1881) V–VI	He	Bor-Ar	67 ± 17	332 ± 64	<0.05
*Scolecithricella minor* (Brady, 1883)	Om	Bor-Ar	-	8 ± 8	na
**Medusae**			8 ± 5	31 ± 12	0.562
*Aeginopsis laurentii* Brandt, 1838	Cr	Ar	-	2 ± 2	na
*Aglantha digitale* (Müller, 1776)	Cr	Bor	-	6 ± 3	na
*Euphysa flammea* (Linko, 1905)	Cr	Bor	5 ± 3	9 ± 3	0.341
*Euphysa* spp. juv.	Cr	Bor	3 ± 2	14 ± 4	<0.05
**Meroplanktonic larvae**					
Cirripedia cypris larvae	Om	Mx	23 ± 23	-	na
Cirripedia nauplii	Om	Mx	1332 ± 318	19,872 ± 10820	0.394
Echinoidea (echinopluteus larvae)	Om	Mx	432 ± 263	524 ± 213	0.793
Gastropoda larvae	Om	Mx	-	236 ± 67	na
Ophiuroidea (ophiopluteus larvae)	Om	Mx	53 ± 30	395 ± 161	0.180
Polychaeta larvae	Om	Mx	1648 ± 484	5108 ± 1884	0.106
*Chionoecetes opilio* (Fabricius, 1788) larvae	Om	Bor-Ar	17 ± 8	237 ± 106	<0.05
*Pagurus* spp. zoea	Om	Bor-Ar	14 ± 12	2 ± 2	0.589
*Lithodes maja* (Linnaeus, 1758) zoea	Om	Bor-Ar	-	2 ± 2	na
*Sabinea spp.* larvae	Om	Bor-Ar	-	2 ± 2	na
*Eualus gaimardi* (H. Milne-Edwards, 1837) larvae	Om	Bor-Ar	-	2 ± 2	na
**Euphausiids**			19,802 ± 6908	3072 ± 947	<0.05
*Meganyctyphanes norvegica* (M. Sars, 1857)	He	Bor	-	2 ± 2	na
*Thysanoessa inermis* (Krøyer, 1846)	He	Bor	5 ± 2	2 ± 2	0.394
*Thyssanoessa raschii* (M. Sars, 1864)	He	Bor-Ar	2 ± 2	5 ± 3	0.589
*Thyssanoessa* spp. calyptopis	He	Bor-Ar	636 ± 582	40 ± 23	0.589
*Thyssanoessa* spp. nauplii	He	Bor-Ar	19,159 ± 6322	3023 ± 917	<0.05
**Hyperiids**			2 ± 2	64 ± 41	<0.05
*Themisto abyssorum* Boeck, 1870	Cr	Bor	-	19 ± 10	na
*Themisto libellula* Lichtenstein, 1822	Cr	Ar	-	36 ± 25	na
*Themisto* juv.	Cr		2 ± 2	9 ± 6	0.589
**Appendicularia**			329 ± 237	23,505 ± 7342	<0.05
*Fritillaria borealis* Lohmann, 1896	Om	Bor-Ar	274 ± 182	21,050 ± 5987	<0.05
*Oikopleura* juv.	Om	Bor-Ar	33 ± 33	2345 ± 1317	<0.05
*Oikopleura vanhoeffeni* Lohmann, 1896	Om	Bor-Ar	22 ± 22	110 ± 38	0.069
**Ctenophora**			5 ± 3	16 ± 10	0.634
*Beroe cucumis* Fabricius, 1780	Cr	Bor-Ar	-	5 ± 3	na
*Mertensia ovum* (Fabricius, 1780)	Cr	Ar	5 ± 3	11 ± 7	0.699
**Others**			120 ± 61	60 ± 34	0.387
*Boroecia borealis* (Sars, 1866)	Om		12 ± 12	-	na
*Clione limacina* (Phipps, 1774) larvae	Cr	Bor-Ar	-	3 ± 3	na
*Limacina helicina* Phipps, 1774 larvae	He	Bor-Ar	-	8 ± 8	na
*Limacina helicina* Phipps, 1774	He	Bor-Ar	33 ± 19	22 ± 9	0.616
*Parasagitta elegans* (Verrill, 1873)	Cr	Bor-Ar	73 ± 28	25 ± 12	0.240
*Tomopteris* spp.	He	Bor	2 ± 2	-	na
Pisces larvae	Cr	Mx	-	2 ± 2	na
**Parameters**					
Total abundance			73 ± 14	171 ± 24	<0.05
Total biomass			846 ± 179	2935 ± 1358	<0.05
J’			0.6 ± 0.02	0.64 ± 0.01	0.202
H’(log_e_)			1.85 ± 0.05	2.17 ± 0.03	<0.001
H’(log_2_)			2.67 ± 0.07	3.13 ± 0.05	<0.001
Temp			0.89 ± 0.27	0.22 ± 0.13	<0.05
Sal			34.70 ± 0.02	34.81 ± 0.03	<0.05
Chla			2.41 ± 0.49	1.21 ± 0.20	<0.05

Note. na—no analysis. I–IV—copepodites I–IV; V–VI—copepodites V and adults. Trophic status. He—herbivorous; Om—omnivorous; Cr—carnivorous. Note that some taxa (e.g., *Calanus* spp. and *Thyssanoessa* spp.) may change their food preference in relation to environmental conditions. We indicate main trophic strategy for each mesozooplankton group. Biogeography: Bor—boreal; Ar—Arctic; Bor-Ar—Boreal-Arctic; Cs—cosmopolitan; Mx- mixed group.

**Table 3:** Spearman’s rank correlation coefficients between environmental variables and the abundance of common mesozooplankton groups in the eastern Barents Sea in May 2016. Bold font indicates significant coefficients after Holm–Bonferroni correction (*p* < 0.05): Lat—latitude; Long—longitude; Depth—depth of sampling, m; Temp—mean temperature in sampling layer, °C; Sal—mean salinity in sampling layer; Chla—surface chlorophyll *a* concentration in sampling layer, mg m^−3^.

Group	Lat	Long	Depth	Temp	Sal	Chla
Copepoda	**0.71**	**0.80**	**0.68**	**−0.60**	**0.65**	**−0.67**
Medusae	0.56	0.49	−0.01	**−0.77**	0.21	**−0.66**
Meroplankon	0.37	0.15	−0.21	−0.49	−0.02	−0.51
Euphausiids	**−0.79**	−0.54	−0.01	0.13	**–0.66**	0.14
Hyperiids	0.56	**0.77**	**0.62**	−0.22	**0.63**	−0.26
Appendicularia	**0.68**	**0.58**	0.14	**−0.59**	0.43	**−0.57**
Total	0.56	**0.62**	0.54	**−0.83**	0.35	**−0.86**

## Data Availability

The data are available on request from the corresponding author.
